# Supplementary figures and images for: Disrupted reinforcement learning during post-error slowing in ADHD
Source: PLoS One. 2019 Feb 20;14(2):e0206780. doi: 10.1371/journal.pone.0206780 (PMC6382150; doi:10.1371/journal.pone.0206780)

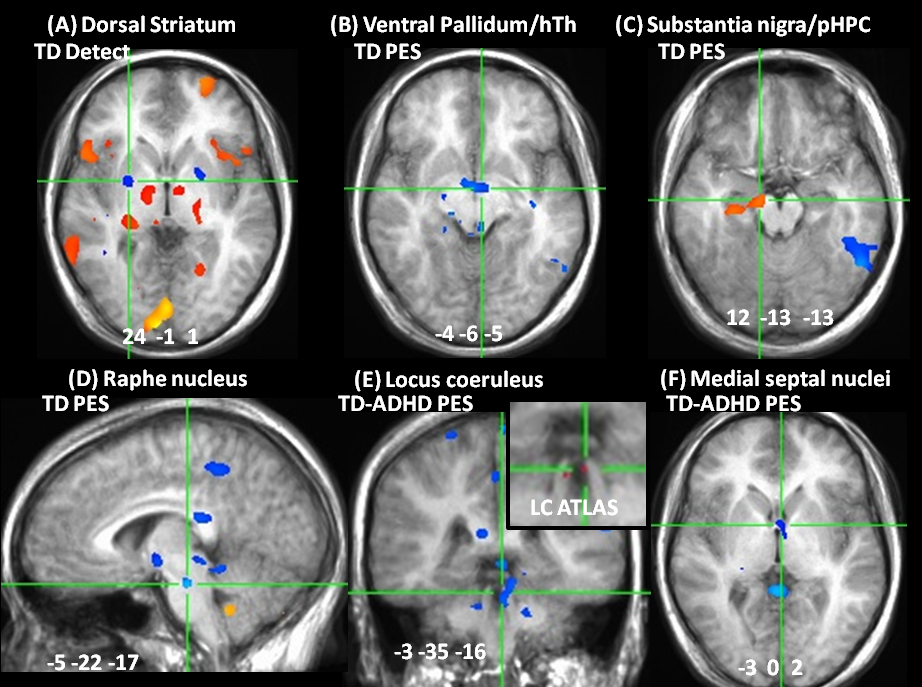

Supplement: S1 Fig — (A) Deactivation of dorsal striatum in TD adolescents during error detection (TD Detect). (B) Deactivation of ventral pallidum in TD during post-error slowing (TD PES). (C) Activation of substantia nigra in TD during post-error slowing (TD PES). (D) Deactivation of raphe nucleus in TD adolescents during post-error slowing (TD PES). (E) Negative group difference in locus coeruleus during post-error slowing (TD-ADHD PES; inset shows locus coeruleus (LC) atlas in red). (F) Negative group difference during post-error slowing in medial septal nuclei (TD-ADHD PES). Activation maps portray percent BOLD estimates after whole brain correction (red/yellow = activation, blue = deactivation). Locations in Talairach space, portrayed in radiological space (left = right); hTh = hypothalamus; pHPC = parahippocampus. (TIF) [file pone.0206780.s001.tif]

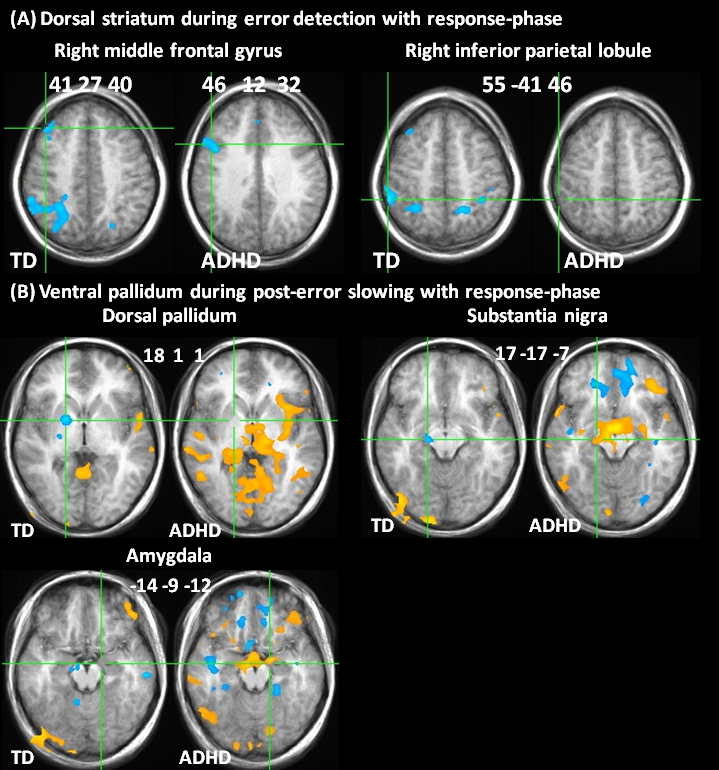

Supplement: S2 Fig — (A) Correlations of dorsal striatum during error detection with right middle frontal gyrus and inferior parietal lobule during response-phases. (B) Correlations of ventral pallidum with dorsal pallidum, substantia nigra and amygdala during error detection. Correlation maps portray B1 estimates after whole brain correction (red/yellow = positive, blue = negative correlation). Locations in Talairach space, portrayed in radiological space (left = right). (TIF) [file pone.0206780.s002.tif]

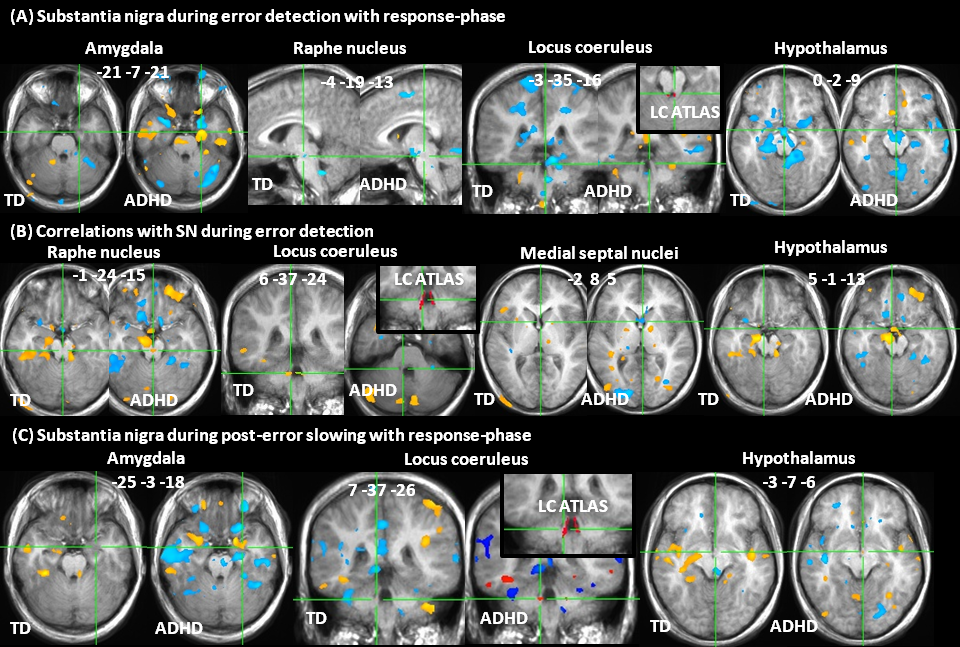

Supplement: S3 Fig — Correlation of substantia nigra during error detection with response-phase (A) and with error detection maps (B). Correlation of substantia nigra during post-error slowing with post-error slowing (C) and response-phase (D) maps. Correlation maps portray B1 estimates after whole brain correction (red/yellow = positive, blue = negative correlation). Locations in Talairach space, portrayed in radiological space (left = right); LC ATLAS = insets showing locus coeruleus atlas in red. (TIF) [file pone.0206780.s003.tif]

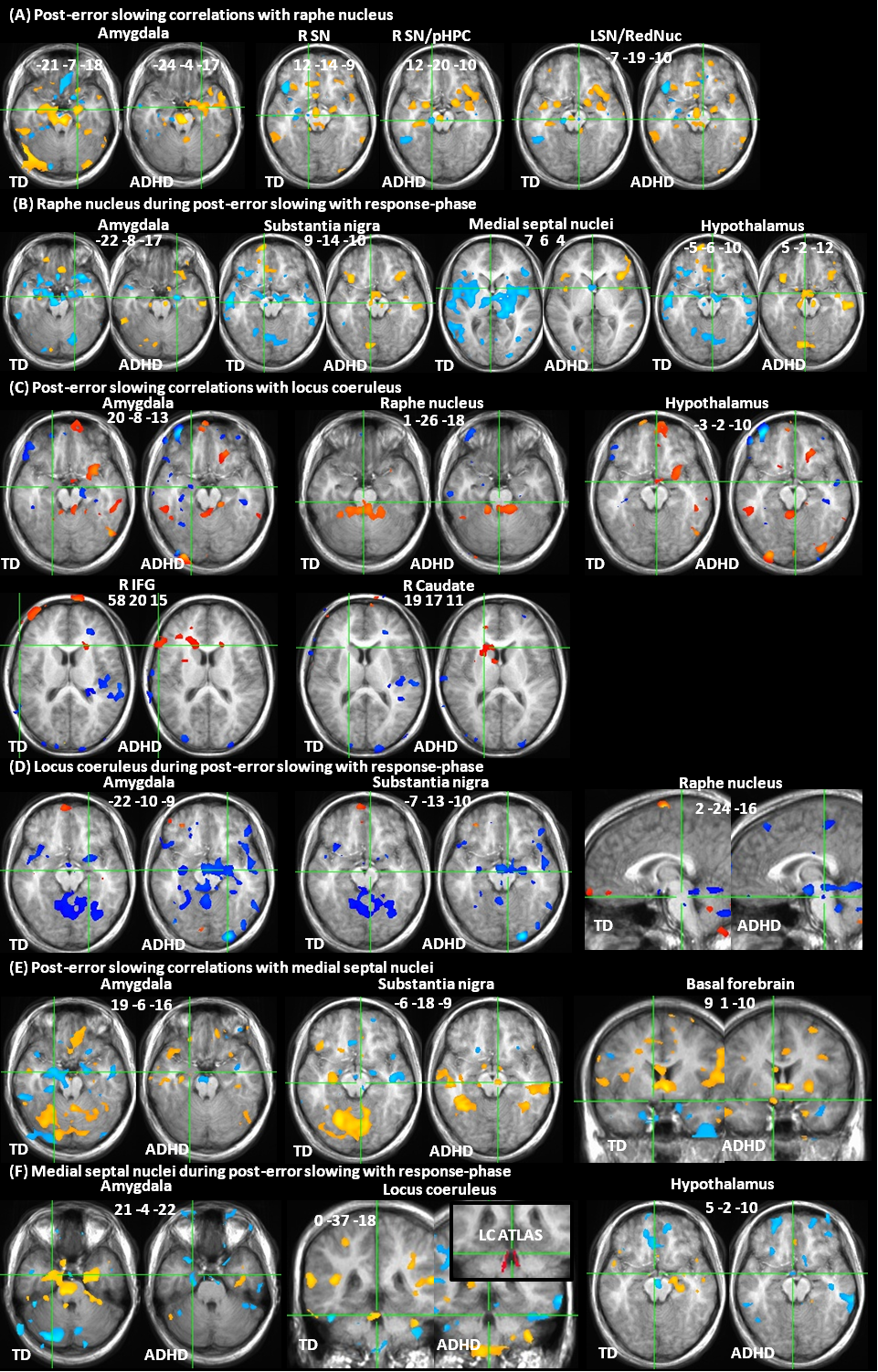

Supplement: S4 Fig — (A) Correlation of raphe nucleus during post-error slowing with post-error slowing (A) and response-phase maps (B). Correlation of locus coeruleus activity during post-error with post-error slowing (C) and with response-phase maps (D). Correlation of medial septal nuclei during post-error slowing with post-error slowing (E) and response-phase maps (F). Correlation maps portray B1 estimates after whole brain correction (red/yellow = positive, blue = - negative correlation). Locations in Talairach space, portrayed in radiological space (left = right). (TIF) [file pone.0206780.s004.tif]

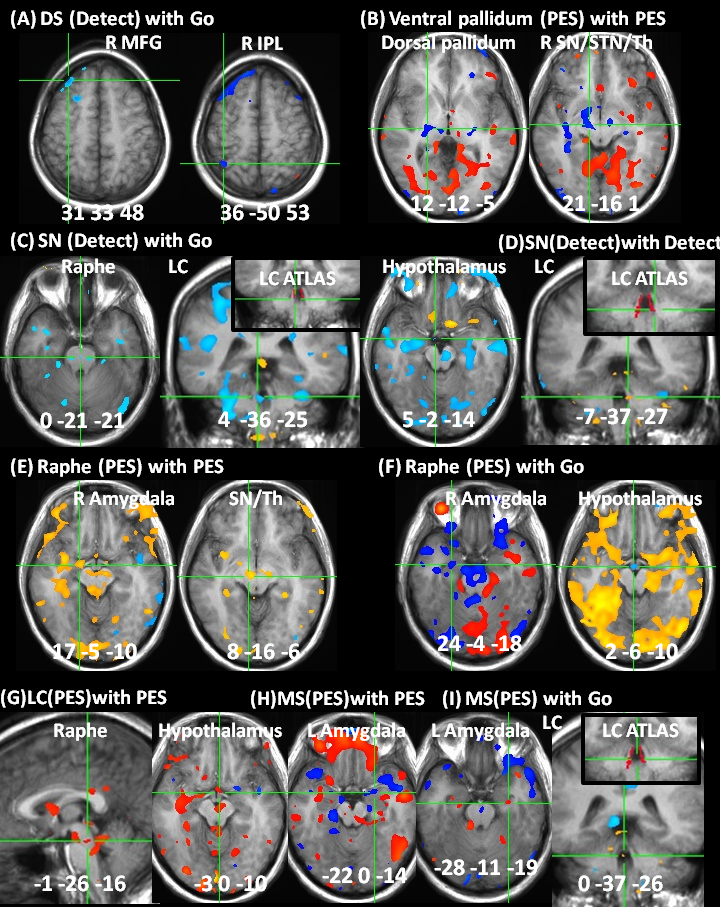

Supplement: S5 Fig — (A) Correlation of dorsal striatum (DS) during error detection (Detect) with response-phase (Go) activities in right middle frontal gyrus (R MFG) and inferior parietal lobule (IPL). (B) Correlation of ventral pallidum with dorsal pallidum and substantia nigra (SN) during post-error slowing (PES). (C) Correlation of SN during Detect with Go activity in raphe nucleus, locus coeruleus (LC) and hypothalamus. (D) SN correlation with LC during Detect. (E) Raphe correlation with amygdala and SN during PES. (F) Correlation of raphe nucleus during PES with amygdala and hypothalamus during Go. (G) Correlation of LC with raphe nucleus and hypothalamus during PES. (H) Correlation of medial septal nuclei (MS) with amygdala during PES. (I) Correlation of MS during PES with LC during Go. Correlation maps (B1 estimates) were whole-brain corrected except where subthreshold (B ii,F i, H). Color bars depict Z-score range. Locations in Talairach space, portrayed in radiological space (left = right). (TIF) [file pone.0206780.s005.tif]

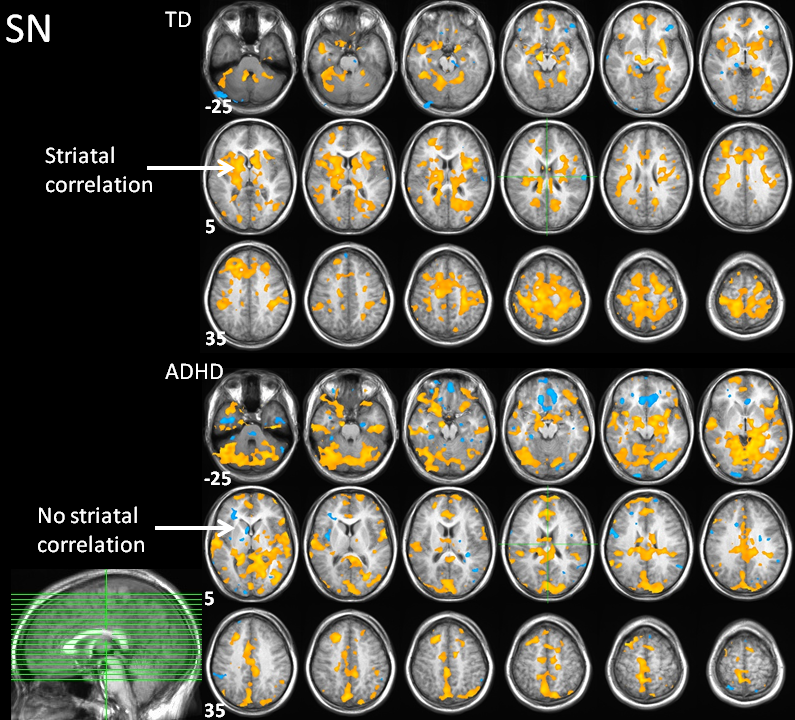

Supplement: S6 Fig — Whole brain corrected correlations (red/yellow = positive, blue = negative correlation) with SN seed during post-error slowing. In TD, SN correlated with bilateral limbic, striatal and neocortical regions, consistent with known ascending DA pathways. ADHD showed stronger correlations in posterior networks and negative correlations were present in rostral ACC. Striatal correlations were absent in ADHD, indicating a lack of nigrostriatal influence during post-error slowing. Numbers indicate slice locations in Talairach coordinates (ascending in 5mm increments), portrayed in midline sagittal plane at bottom left. (TIF) [file pone.0206780.s006.tif]

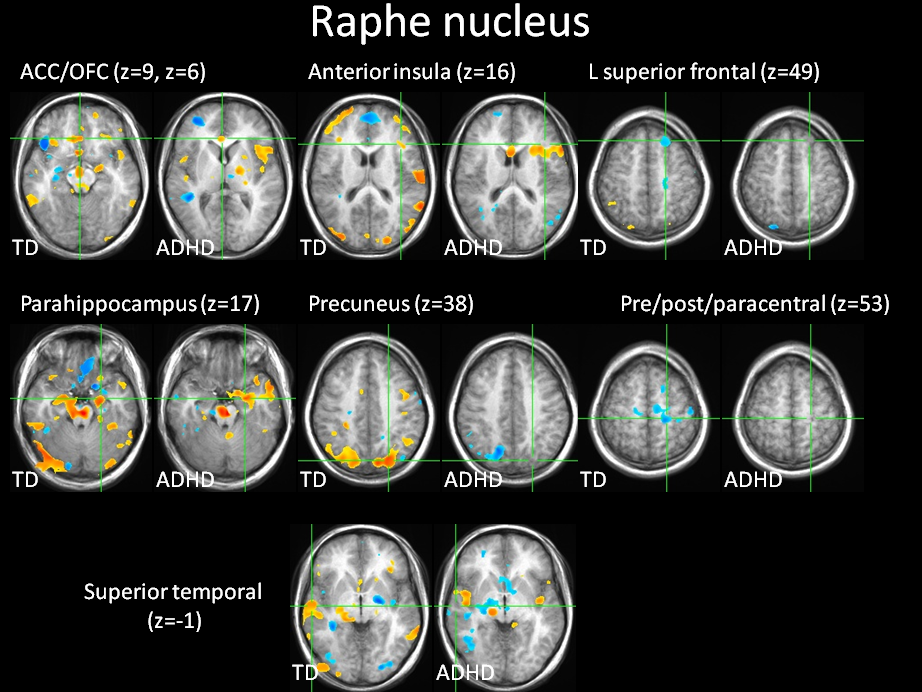

Supplement: S7 Fig — Whole brain corrected correlations (red/yellow = positive, blue = negative correlation) with raphe nucleus seed during post-error slowing in the same regions and direction (positive/negative) as in [45]. Correlations were weaker or absent in ADHD except in anterior insula, where correlations were stronger than in TD. z = slice location in Talairach coordinates. (TIF) [file pone.0206780.s007.tif]
